# Induction of Apoptosis by Luteolin Involving Akt Inactivation in Human 786-O Renal Cell Carcinoma Cells

**DOI:** 10.1155/2013/109105

**Published:** 2013-02-05

**Authors:** Yen-Chuan Ou, Yu-Hsiang Kuan, Jian-Ri Li, Shue-Ling Raung, Chung-Chiang Wang, Yu-Yeh Hung, Chun-Jung Chen

**Affiliations:** ^1^Division of Urology, Taichung Veterans General Hospital, Taichung 407, Taiwan; ^2^Department of Pharmacology, School of Medicine, Chung-Shan Medical University, Taichung 402, Taiwan; ^3^Department of Education and Research, Taichung Veterans General Hospital, Taichung 407, Taiwan; ^4^Center for General Education, Tunghai University, Taichung 407, Taiwan; ^5^Institute of Biomedical Sciences, National Chung Hsing University, Taichung 402, Taiwan; ^6^Graduate School of Nursing, Hung-Kuang University, Taichung 433, Taiwan

## Abstract

There is a growing interest in the health-promoting effects of natural substances obtained from plants. Although luteolin has been identified as a potential therapeutic and preventive agent for cancer because of its potent cancer cell-killing activity, the molecular mechanisms have not been well elucidated. This study provides evidence of an alternative target for luteolin and sheds light on the mechanism of its physiological benefits. Treatment of 786-O renal cell carcinoma (RCC) cells (as well as A498 and ACHN) with luteolin caused cell apoptosis and death. This cytotoxicity was caused by the downregulation of Akt and resultant upregulation of apoptosis signal-regulating kinase-1 (Ask1), p38, and c-Jun N-terminal kinase (JNK) activities, probably via protein phosphatase 2A (PP2A) activation. In addition to being a concurrent substrate of caspases and event of cell death, heat shock protein-90 (HSP90) cleavage might also play a role in driving further cellular alterations and cell death, at least in part, involving an Akt-related mechanism. Due to the high expression of HSP90 and Akt-related molecules in RCC and other cancer cells, our findings suggest that PP2A activation might work in concert with HSP90 cleavage to inactivate Akt and lead to a vicious caspase-dependent apoptotic cycle in luteolin-treated 786-O cells.

## 1. Introduction

There is growing evidence of and interest in the health benefits of foods of plant origin due to their diversity of biological activities. Medicinal plants, plant extracts, and isolated secondary metabolites have traditionally been used to treat several clinical diseases. Epidemiological studies have shown that the consumption of vegetables, fruits, and tea may help to delay or prevent carcinogenesis. Their preventive and therapeutic roles in cancer have been attributed to their high polyphenol content, particularly flavonoids [1, 2]. Flavonoids are known to possess various antineoplastic properties, and among these their proapoptotic effects have been extensively studied [3]. Luteolin (3′,4′,5′,7′-tetrahydroxyflavone), a polyphenolic compound found in plants such as celery, green peppers, perilla leaf, and chamomile tea, belongs to the flavone subclass of flavonoids. Luteolin possesses cancer cell-killing activity as well as the ability to resensitize cancer cells to chemotherapeutic or biotherapeutic agents. The antineoplastic activity of luteolin is attributed to its ability to induce DNA damage, cell cycle arrest, and apoptosis and to suppress angiogenesis and cell survival capacity [4–11]. At present, the antineoplastic property of luteolin has been evaluated mainly with respect to its ability to induce apoptosis [4, 5, 7, 11]. It is recognized that the antineoplastic activity of luteolin on the diversity of cancer cells has been linked to its effect on numerous intracellular biochemical pathways critical in regulating cell survival and apoptosis. Despite the well-documented antineoplastic potential of luteolin, relatively little is known about the involved signaling molecules in transducing its proapoptotic action.

Renal cell carcinoma (RCC) is the most frequent and lethal malignant tumor of the kidney in adults and exhibits highly vascularized characteristics. Most symptomatic patients present with advanced metastatic disease, and thus have a poor prognosis. Until recently, therapeutic options for unresectable and/or metastatic RCC were limited, as RCC is typically refractory to traditional chemotherapy, hormonal therapy, and radiation therapy. Immunotherapy, including interleukin-2 and interferon-*α*, provides only limited benefit [12, 13]. Nevertheless, new insights into the molecular signature of RCC have led to novel therapeutic strategies for the management of advanced disease. Indeed, clear cell RCC is characterized by the inactivation of von Hippel Lindau protein (VHL) and a constitutive activation of hypoxia-inducible factor (HIF) leading to consequences of overactivation of phosphatidylinositol-3 kinase (PI3K) and mammalian target of rapamycin (mTOR) and overproduction of vascular endothelial growth factor (VEGF). Recently, novel agents for RCC targeting overactivated pathways have been developed or actively investigated, such as receptor tyrosine kinase inhibitors (sunitinib, sorafenib, axitinib, pazopanib), anti-VEGF monoclonal antibody (bevacizumab), mTOR inhibitors (everolimus, temsirolimus), and PI3K/mTOR dual inhibitor (NVP-BEZ235) [14–19]. Although they have been shown to be beneficial in patients with advanced RCC, the effect is insufficient and is associated with significant adverse effects. It is therefore necessary to discover novel targets for the treatment of RCC and develop alternative agents for patients who are not responsive and/or intolerant to these therapies.

Luteolin is reported to inhibit the development of a series of solid tumors via apoptosis [4–11]; however, the precise mechanism of its effect remains to be fully elucidated. Investigations of the signal transduction pathways responsible for such apoptotic mediator induction leading to cell survival or apoptosis have focused on the mitogen-activated protein kinase (MAPK) and Akt/protein kinase B pathway. It has been recognized that the activation of the p38 and c-Jun N-terminal kinase (JNK) pathway is involved in stress stimuli-induced apoptosis, whereas extracellular signal-regulated kinase (ERK) and Akt have been shown to protect cells from apoptosis [20–22]. There is growing evidence showing that the activation of p38 and JNK plays a major role in triggering RCC apoptosis [23, 24]. These studies indicate that both p38 and JNK warrant further investigation as potential targets for prevention and treatment of human cancers. Evidence suggests that signaling molecules such as p38 and JNK are crucial for induction of apoptosis caused by luteolin [4, 7, 10, 11]. Currently, the effect of luteolin on human RCC has not been addressed. In this study, we investigated whether luteolin possesses proapoptotic activities against RCC and whether MAPKs and Akt are involved in luteolin-induced apoptosis and, if this proved to be the case, to identify the potential upstream regulatory effectors.

## 2. Materials and Methods

### 2.1. Cell Cultures

Human RCC cell lines, 786-O (ATCC CRL1932), A498 (ATCC HTB-44), and ACHN (ATCC CRL-1611) were cultured in Dulbecco's modified Eagle medium (DMEM) supplemented with 10% fetal bovine serum (FBS), 100 U/mL penicillin, and 100 *μ*g/mL streptomycin and were maintained in a humidified incubator with 5% CO_2_. When experimenting, cells were switched to DMEM containing 2% FBS.

### 2.2. Cell Viability

Cell viability was assessed by the measurement of formazan production after the addition of 3-(4,5-dimethylthiazol-2-yl)-5-(3-carboxymethoxyphenyl)-2-(4-sulphophenyl)-2H-tetrazolium, inner salt (MTS, Promega, Madison, WI). The number of surviving cells after treatment was determined by measurement of the A_490_ nm of the dissolved formazan product after the addition of MTS for 1 h according to the manufacturer's instructions.

### 2.3. Flow Cytometry Assay

The cell cycle distribution was analyzed by flow cytometry [25]. Briefly, cells were trypsinized, washed with phosphate-buffered saline (PBS), and fixed in 80% ethanol. They were then washed with PBS, incubated with 100 *μ*g/mL RNase at 37°C for 30 min, stained with propidium iodide (50 *μ*g/mL), and analyzed on a FACScan flow cytometer. The percentage of cells in different phases of the cell cycle was analyzed using Cell-FIT software.

### 2.4. Caspase Activity Assay

Caspase activity assay was carried out using a fluorometric protease assay kit following the instructions provided by the manufacturer (BioVision, Mountain View, CA). In brief, cells were homogenized on ice with kit-provided lysis buffer. An aliquot of 50 *μ*L of supernatants was incubated with an equal volume of the reaction buffer containing fluorogenic peptide substrate at 37°C for 1~2 h. Enzymatic release of free fluorogenic moiety was measured by a fluorometer (E_x_ 380 nm and E_m_ 460 nm).

### 2.5. Western Blot

Obtained cell extracts were separated by sodium dodecyl sulfate polyacrylamide gel electrophoresis and electrophoretically transferred to polyvinylidene difluoride membranes. After blocking, the membranes were incubated with the indicated antibodies against: PARP-1, FAK, heat shock protein 90 (HSP90), ERK, phospho-ERK (Thr-202/Tyr-204), p38, phospho-p38 (Thr-180/Tyr-182), JNK, phospho-JNK (Thr-183/Tyr-185), Akt, phospho-Akt (Ser-473) (Santa Cruz Biotechnology, Santa Cruz, CA), apoptosis signal-regulating kinase-1 (Ask1, R&D Systems, Minneapolis, MN), phospho-Ask1 (Ser-83) (GeneTex, Irvine, CA), and *β*-tubulin (Sigma-Aldrich, St. Louis, MO). Then the membranes were incubated with horseradish peroxidase-labeled IgG. The blots were developed using enhanced chemiluminescence Western blotting reagents. The intensity of each signal was determined by a computer image analysis system (IS1000; Alpha Innotech Corporation).

### 2.6. Immunoprecipitation

Cells were washed twice with PBS and harvested in RIPA buffer [26]. Protein A-agarose beads were washed with RIPA buffer and then incubated with antibodies for 1 h at room temperature. After removal of the unbound antibodies, protein extracts (200 *μ*g) were added with gentle shaking and incubated for an additional 4 h at room temperature. Immunoprecipitates were washed with RIPA buffer and then eluted for further analysis.

### 2.7. Phosphatase Assay

After washing with PBS, cells were resuspended with PBS, subjected to three rounds of freeze/thaw, and then sonicated for 10 s. Protein phosphatase 2A (PP2A) activity was measured using a commercially available serine/threonine phosphatase assay kit (Molecular Probes, Eugene, OR). Five micrograms of proteins were added and reacted with preloaded substrates. The generated fluorescent product was determined using a fluorometer (E_x_ 358 nm and E_m_ 452 nm).

### 2.8. Statistical Analysis

The data are expressed as mean values ± standard deviation. Statistical analysis was carried out using one-way analysis of variance, followed by Dunnett's test to assess the statistical significance between treated and untreated groups. A level of *P* < 0.05 was considered statistically significant.

## 3. Results

### 3.1. Luteolin Reduced Cell Viability and Induced Apoptosis

**Figure 1:**
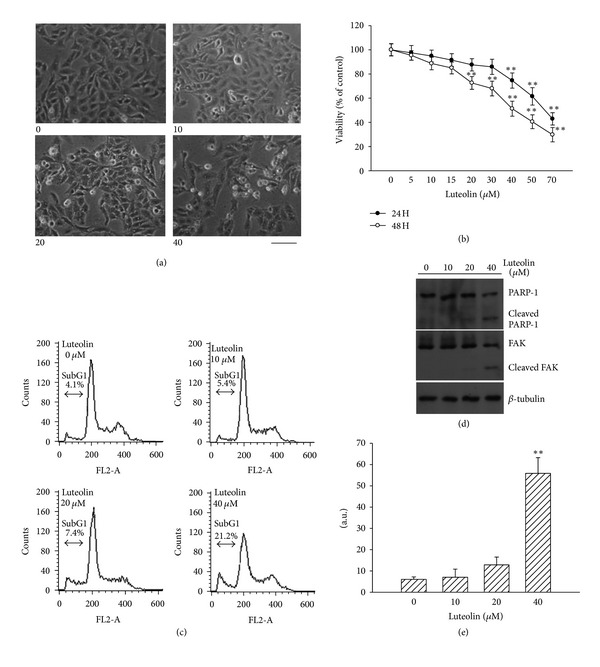
Effects of luteolin on cell viability. 786-O cells were treated with various concentrations of luteolin. Representative images of phase contrast were obtained 24 h after treatment. Scale  bar = 60 *μ*m (a). 786-O cells were treated with various concentrations of luteolin over time. Cell viability was determined by MTS reduction assay (b). 786-O cells were treated with various concentrations of luteolin for 24 h. The cells were harvested and processed by flow-cytometric analysis. The percentage of subG1 population is shown (c). Protein extracts were isolated and subjected to Western blot analysis with indicated antibodies. One of four independent experiments is shown (d). Protein extracts were isolated and subjected to fluorogenic caspase-3 assay. The intensity of fluorescent signals was expressed as arbitrary unit (e). **P < 0.01 versus medium control, *n* = 4.

To determine the effect of luteolin on human RCC cell viability, 786-O cells were treated with luteolin (L9283, Sigma-Aldrich, St. Louis, MO). Distinctive morphological changes, including cellular rounding, shrinkage, membrane blebbing, and separation from neighboring cells were observed for 786-O cells treated with increasing concentrations of luteolin (Figure 1(a)). MTS reduction assay revealed that luteolin reduced cell viability in a time- and concentration-dependent manner (Figure 1(b)). The calculated IC_50_ of luteolin was ~64 *μ*M at 24 h and ~45 *μ*M at 48 h. These action concentrations were similar to those did in human breast adenocarcinoma cells [11]. To understand the mechanism by which luteolin caused viability loss in 786-O cells, several experiments were carried out involving apoptosis. In parallel with viability loss, the generation of abnormal diploid DNA content (subG1) (Figure 1(c)), proteolytic cleavage of PARP-1 and FAK (Figure 1(d)) and elevation of caspase-3 activity (Figure 1(e)) were observed in luteolin-treated cells. Taken together, our findings indicate that the viability loss of 786-O cells caused by luteolin was associated with caspase-related apoptotic injury.

### 3.2. MAPKs and Akt Involved in Luteolin-Induced Apoptosis

**Figure 2:**
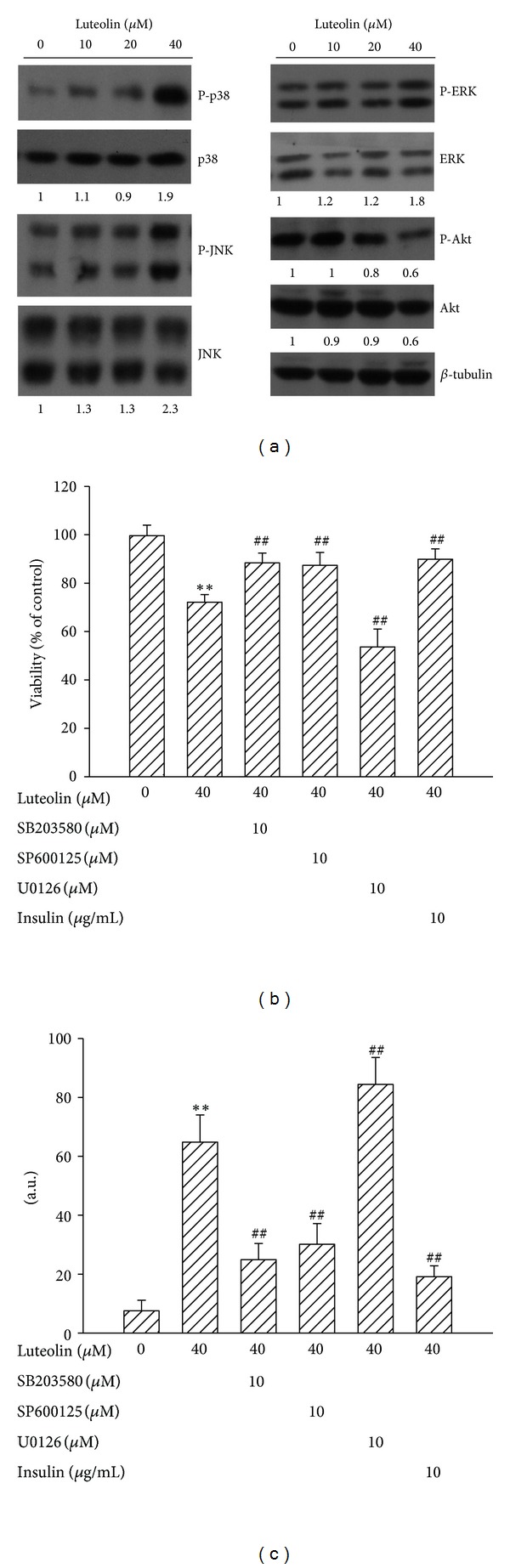
Effects of luteolin on MAPKs and Akt. 786-O cells were treated with various concentrations of luteolin for 24 h. Protein extracts were isolated and subjected to Western blot analysis with indicated antibodies. One of four independent experiments is shown (a). 786-O cells were pretreated with medium, SB203580, SP600125, U0126, or insulin for 1 h and then were treated with luteolin for additional 24 h. Cell viability was determined by MTS reduction assay (b). Protein extracts were isolated and subjected to fluorogenic caspase-3 assay. The intensity of fluorescent signals was expressed as arbitrary unit (c). **P < 0.01 versus medium control and ^##^ *P* < 0.01 versus luteolin control, *n* = 4.

**Figure 3:**
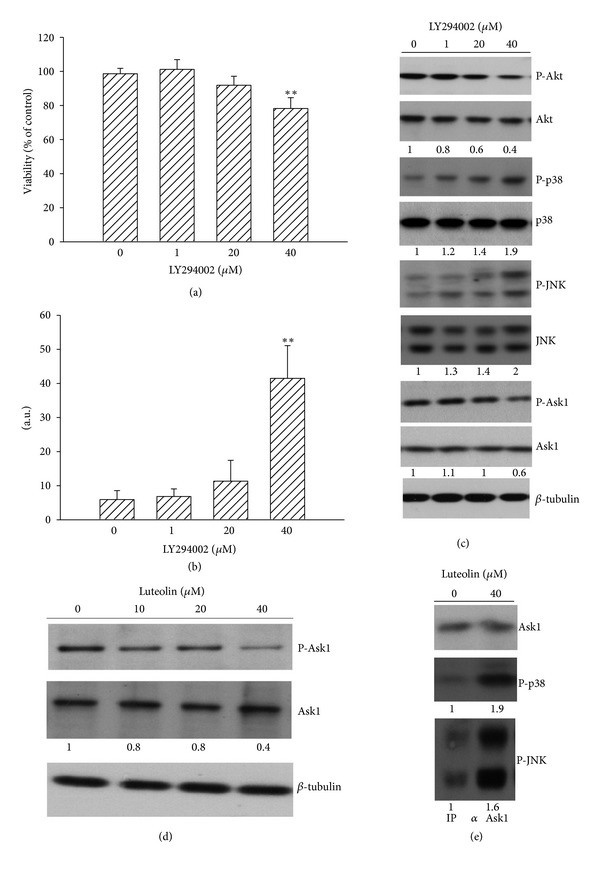
Role of Akt. 786-O cells were treated with various concentrations of LY294002 for 24 h. Cell viability was determined by MTS reduction assay (a). Protein extracts were isolated and subjected to fluorogenic caspase-3 assay. The intensity of fluorescent signals was expressed as arbitrary unit. **P < 0.01 versus medium control, *n* = 4 (b). Protein extracts were isolated and subjected to Western blot analysis with indicated antibodies. One of four independent experiments is shown (c). 786-O cells were treated with various concentrations of luteolin for 24 h. Protein extracts were isolated and subjected to Western blot analysis with indicated antibodies. One of four independent experiments is shown (d). Proteins obtained from medium- and luteolin (40 *μ*M)-treated cells were immunoprecipitated by anti-Ask1 antibody, and the immunoprecipitates were subjected to Western blot analysis with indicated antibodies. One of four independent experiments is shown (e).

There is evidence showing that cellular signaling molecules such as MAPKs and Akt play a crucial role in cell apoptosis [20–22]. To examine whether MAPKs and Akt play roles in luteolin-induced 786-O cell death, biochemical and pharmacological approaches were used. Increasing concentrations of luteolin increased protein phosphorylations in p38, JNK, and ERK but decreased protein phosphorylation in Akt (Figure 3(a)). Treatment of 786-O cells with SB203580 (p38 inhibitor), SP600125 (JNK inhibitor), or insulin (Akt activator) led to a decrease in luteolin-induced viability loss (Figure 2(b)) and caspase-3 activation (Figure 2(c)). In contrast, U0126 (ERK inhibitor) exacerbated those luteolin-induced alterations (Figures 2(b) and 2(c)). Since our findings suggest the potential active association of p38 and JNK activation and Akt inactivation in luteolin-induced 786-O cell death, their potential crosstalk was further examined. Pharmacological inhibition of Akt by LY294002 was able to decrease 786-O cell viability (Figure 3(a)), and elevate caspase-3 activity (Figure 3(b)). The inactivation of Akt was accompanied by increased p38 and JNK phosphorylation (Figure 3(c)). Evidence shows that Ask1 is a potential modulatory molecule linking Akt and p38/JNK [22, 27, 28]. Decreased Ask1 phosphorylation in serine 83, a residue which negatively regulates Ask1 activity, was observed in LY294002-treated cells (Figure 3(c)). This decreased Ask1 phosphorylation was also observed in luteolin-treated 786-O cells (Figure 3(d)). The interplay between Ask1 and p38 and JNK was confirmed by functional protein complexes. Immunoprecipitation and Western blot assay revealed an increased protein complex between activated p38/JNK and Ask1 in luteolin-treated cells (Figure 3(e)). There was no signal detected after the precipitation with control immunoglobulin (data not shown). These findings suggest a crucial role of Akt in the decision of 786-O cell viability, and a mutual interaction between Akt and p38/JNK and Akt inhibition could lead to cell death that strongly correlates with activation of p38 and JNK after luteolin treatment.

### 3.3. HSP90 Involved in Luteolin-Induced Akt Inactivation

**Figure 4:**
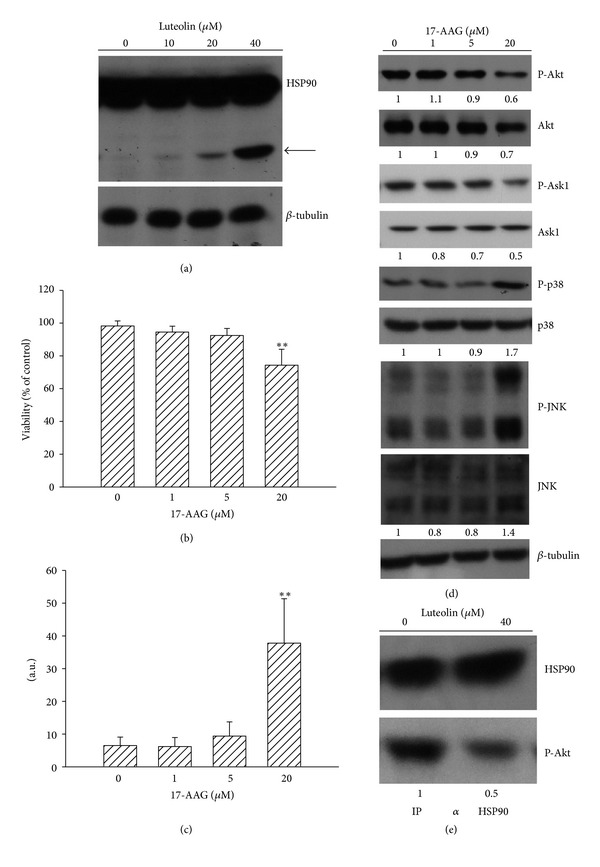
Role of HSP90. 786-O cells were treated with various concentrations of luteolin for 24 h. Protein extracts were isolated and subjected to Western blot analysis with indicated antibodies. One of four independent experiments is shown. An additional band of HSP90 was indicated by arrow (a). 786-O cells were treated with various concentrations of 17-AAG for 24 h. Cell viability was determined by MTS reduction assay (b). Protein extracts were isolated and subjected to fluorogenic caspase-3 assay. The intensity of fluorescent signals was expressed as arbitrary unit. **P < 0.01 versus medium control, *n* = 4 (c). Protein extracts were isolated and subjected to Western blot analysis with indicated antibodies. One of four independent experiments is shown (d). Proteins obtained from medium- and luteolin (40 *μ*M)-treated cells were immunoprecipitated by anti-HSP90 antibody, and the immunoprecipitates were subjected to Western blot analysis with indicated antibodies. One of four independent experiments is shown (e).

Since Akt inactivation plays a role in transmitting luteolin's signal to activate p38/JNK through Ask1 leading to cell death, we then sought to determine which upstream signaling molecules involved in the modulation of Akt are changed in luteolin-treated 786-O cells. One of the most extensively characterized regulators of the Akt signaling pathway is cellular chaperone protein HSP90 [22, 29], and therefore we attempted to determine its alterations and contribution to cell survival in luteolin-treated 786-O cells.  Figure 4(a) shows the appearance of an additional HSP90 protein band in 786-O cells treated with luteolin. As shown in other relevant studies [29, 30], this proteolytic cleavage of HSP90 appeared to demonstrate its inactivation because of the degradation of client protein Akt (Figure 2(a)). To elicit the potential role of HSP90 inhibition in the decision of 786-O cell viability, pharmacological inhibitor was used. As with luteolin, inhibition of HSP90 by 17-N-allylamino-17-demethoxygeldanamycin (17-AAG) also led to a decrease of cell viability (Figure 4(b)) and an increase of caspase-3 activity (Figure 4(c)). There was a decreased protein content of Akt and phosphorylation was observed in 17-AAG-treated cells. These changes were accompanied by decreased phosphorylation of Ask1 in serine 83 and increased phosphorylation of p38 and JNK (Figure 4(d)). In comparison with medium control, immunoprecipitation and Western blot assay revealed a decreased association of activated Akt and HSP90 after luteolin treatment (Figure 4(e)). There was no signal detected after the precipitation with control immunoglobulin (data not shown). These findings suggest that HSP90 inhibition by 17-AAG might trigger intracellular signaling cascades similar to those caused by luteolin thereby inducing 786-O cell apoptosis.

### 3.4. PP2A Involved in Luteolin-Induced Akt Inactivation

**Figure 5:**
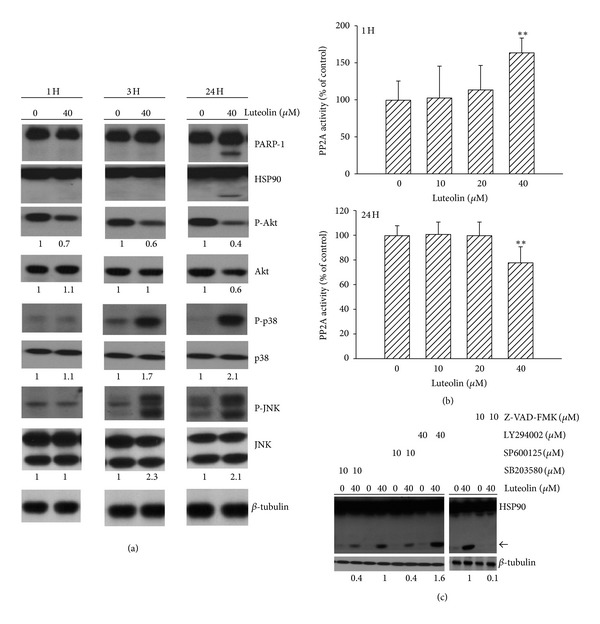
Effects of agents on HSP90 cleavage. 786-O cells were treated with luteolin (0 and 40 *μ*M) for 1 h, 3 h, and 24 h. Protein extracts were isolated and subjected to Western blot analysis with indicated antibodies. One of four independent experiments is shown (a). 786-O cells were treated with various concentrations of luteolin for 1 h and 24 h. Protein extracts were isolated and subjected to enzymatic assay for measurement of PP2A. **P < 0.01 versus medium control, *n* = 4 (b). 786-O cells were pretreated with medium, SB203580, SP600125, LY294002, or Z-VAD-FMK for 1 h and then were treated with luteolin for additional 24 h. Protein extracts were isolated and subjected to Western blot analysis with indicated antibodies. An additional band of HSP90 was indicated by arrow. One of four independent experiments is shown (c).

To get a close insight into the association of HSP90 cleavage, Akt inactivation, and p38/JNK activation, temporal profiles of their alterations caused by luteolin were examined (Figure 5(a)). The proteolytic cleavage of PARP-1, evidence of apoptosis, was accompanied by the generation of HSP90 cleavage, decreased Akt phosphorylation, decreased Akt content, increased p38 phosphorylation, and increased JNK phosphorylation at late stage (24 h) after luteolin treatment. Only the decreased Akt phosphorylation, increased p38 phosphorylation, and increased JNK phosphorylation occurred at early stage (3 h). Especially, the level of Akt phosphorylation started to decrease as early as 1 h after luteolin treatment. To identify the cause of rapid Akt dephosphorylation after luteolin treatment, the activity of a selective Akt phosphatase PP2A was measured. Luteolin caused an increase at early stage (1 h) and a decrease at late stage (24 h) of PP2A activity (Figure 5(b)). Since HSP90 still regulated Akt stability and activity and associated apoptosis in 786-O cells, the signature of luteolin-induced HSP90 cleavage was investigated (Figure 5(c)). Pharmacological inhibition of p38 and JNK remarkably attenuated luteolin-induced HSP90 cleavage, whereas the inhibition of Akt augmented it. The broad spectrum caspase inhibitor, Z-VAD-FMK, caused almost complete inhibition in HSP90 cleavage caused by luteolin. These findings suggest that PP2A activation might work in concert with HSP90 cleavage to inactivate Akt and lead to a vicious caspase-dependent apoptotic cycle in luteolin-treated 786-O cells.

### 3.5. HSP90 Inhibitor and Akt Inhibitor Augmented Luteolin-Induced Viability Loss

**Figure 6:**
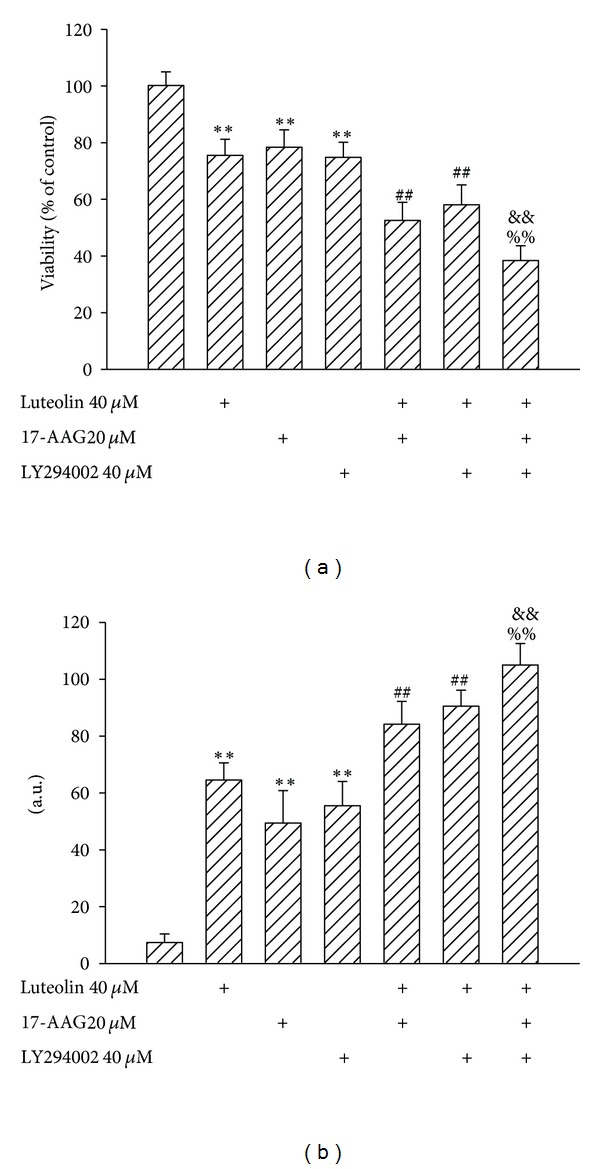
Effects of agents on cell viability. 786-O cells were treated with medium, luteolin, 17-AAG, or LY294002 alone, or in combinations for 24 h. Cell viability was determined by MTS reduction assay (a). Protein extracts were isolated and subjected to fluorogenic caspase-3 assay. The intensity of fluorescent signals was expressed as arbitrary unit (b). **P < 0.01 versus medium control, ^##^ *P* < 0.01 versus luteolin control, ^&&^ *P* < 0.01 versus luteolin/17-AAG, and ^%%^ *P* < 0.01 versus luteolin/LY294002, *n* = 4.

As we had found that cell death caused by luteolin treatment was accompanied by inactivation of Akt and HSP90 and both inhibitors of Akt and HSP90 were capable of inducing cell death, we thought it would be interesting to investigate their combinatory effects on cell survival. 17-AAG and LY294002 caused an additional cytotoxicity in luteolin-treated 786-O cells, respectively. The profound cytotoxicity was observed in cells treated with 17-AAG, LY294002, and luteolin (Figure 6(a)). Similar effects were found in the measurement of caspase-3 activity (Figure 6(b)). These findings show that combinatory inhibition of Akt and HSP90 might promote luteolin-induced cytotoxicity.

### 3.6. Luteolin Reduced Cell Viability and Induced Apoptosis in Other RCC Cell Lines

**Figure 7:**
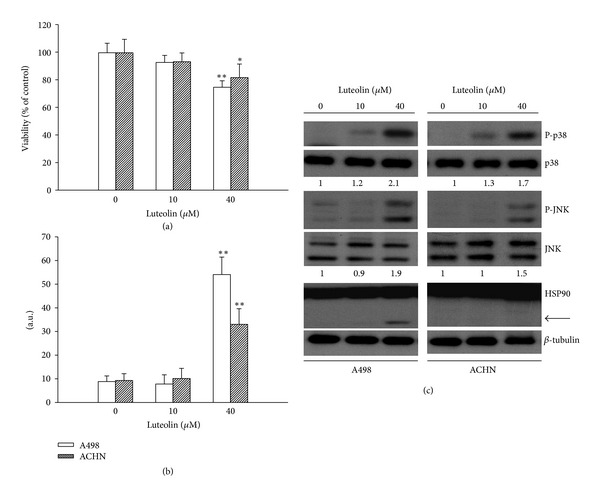
Effects of luteolin on A498 and ACHN cells. A498 and ACHN cells were treated with various concentrations of luteolin for 24 h. Cell viability was determined by MTS reduction assay (a). Protein extracts were isolated and subjected to fluorogenic caspase-3 assay. The intensity of fluorescent signals was expressed as arbitrary unit (b). *P < 0.05 and **P < 0.01 versus medium control, *n* = 4. Protein extracts were isolated and subjected to Western blot analysis with indicated antibodies. One of three independent experiments is shown (c). An additional band of HSP90 was indicated by arrow.

To further demonstrate the effect of luteolin on human RCC in addition to VHL-null renal carcinoma cell line 786-O, A498, another VHL-null renal carcinoma cell line and ACHN VHL wild-type renal carcinoma cell line were tested. As in 786-O cells, luteolin also reduced cell viability (Figure 7(a)), elevated caspase-3 activity (Figure 7(b)), and increased protein phosphorylations in p38 and JNK (Figure 7(c)) in A498 and ACHN cells. Intriguingly, the appearance of an additional HSP90 band was detected in A498 cells but not in ACHN cells (Figure 7(c)). These findings show that luteolin might be a common proapoptotic agent for human RCC and induce cell line-selective mechanism for HSP90 cleavage.

## 4. Discussion

Apoptosis is an important phenomenon in antineoplastic agent-induced tumor cell killing. The reason that RCC fails to respond to traditional cancer therapy could be due to a defect in and/or inactivation of cellular apoptotic machinery. Accumulating evidence suggests that MAPKs and Akt are crucial regulators of apoptosis [20–22]. The results presented in this study provide clear evidence that luteolin exhibits cytotoxic effects on human RCC via an apoptotic mechanism independently on VHL status. The apoptosis caused by luteolin is accompanied by the activation of p38, JNK, and ERK and the inactivation of Akt. The inactivation of Akt and the activation of p38 and JNK contribute to luteolin-induced apoptosis, whereas the activation of ERK seem to buffer the apoptotic burden. Moreover, these individual factors can work in synergy to aggravate cell apoptosis through mutual interaction between Akt, p38, and JNK. Other potential mutual interactions were not addressed in the current study. For example, inactivated Akt might also induce apoptosis through hypophosphorylation of Bad. The results will hopefully highlight the potential targets of Akt, p38, and JNK for therapeutic intervention and the therapeutic potential of luteolin as a novel adjuvant in RCC treatment.

A bulk of evidence suggests that the activation of ERK and Akt signaling increases the cell death threshold. Conversely, the activation of p38 and JNK kinase cascades is generally associated with an enhanced activation of the apoptotic program [20–22]. Many studies have shown that luteolin could activate p38 and JNK leading to cell apoptosis in cancer cells [4, 7, 10, 11]. Consistent with these reports, our results revealed that luteolin also activated p38 and JNK in RCC cells with accompanying induction of apoptosis. Although the p38 and JNK pathways have been implicated as luteolin targets, their upstream regulatory signature for luteolin has not been fully elucidated. Among the stress-activated kinases, Ask1 represents a mitogen-activated protein kinase kinase kinase family member that acts to upstream p38 and JNK. Normally, Ask1 forms a resting conformation characterized by the phosphorylation at the serine-83 amino acid residue and resides in a multimolecular complex associated with several endogenous inhibitory proteins. A variety of stress-related stimuli activate Ask1 by resolving inhibitory folding and dissociating inhibitory proteins, resulting in Ask1 homo-oligomerization and autophosphorylation (threonine-838)-dependent activation. Activated Ask1 leads to the recruitment/activation of downstream effectors. Evidence suggests that Akt is one of regulatory molecules involved in inhibiting Ask1 via phosphorylation at serine-83 [27, 31, 32]. Here, we report that the inactivation of Akt could resolve the auto-inhibition of Ask1 leading to the consequences of Ask1 activation and its downstream effectors, p38 and JNK in luteolin-treated 786-O cells. This hypothesis is partially supported by the decreased Akt and Ask1 serine-83 phosphorylation and the increased protein complexes between activated p38/JNK and Ask1 after luteolin treatment. Furthermore, pharmacological inhibition of Akt by LY294002 caused a reduction of Akt phosphorylation which was accompanied by decreased Ask1 serine-83 phosphorylation, increased p38 and JNK phosphorylation, and apoptosis. The activation of Ask1-p38/JNK pathways was demonstrated in stimuli-induced RCC apoptosis [33]. Taken together, our findings indicate the importance of Ask1-p38/JNK pathways in determining cell apoptosis in RCC and the potential involvement of Ask1-p38/JNK pathways in luteolin-induced 786-O cell apoptosis.

Our data showed that Akt signaling was inhibited by luteolin, as manifested by decreased phosphorylation levels. Evidence indicates that flavonoids inactivate phosphorylation events by competing with the ATP binding site [34]. Therefore, the interference of kinase reaction might be a means by which luteolin downregulates Akt. A growing body of evidence shows Akt activity may be modulated through an alternative mechanism, namely, via HSP90. HSP90, an ATPase-directed chaperone, plays a crucial role in various cellular processes including signal transduction, protein degradation, protein folding, maturation of client proteins, and protein trafficking. Most of the client proteins, which require HSP90 for their conformational maturation, are kinases and signaling molecules of oncogenic potential; therefore, HSP90 occupies a unique role for malignant transformation [22, 29, 30, 35–37]. Among the client proteins, Akt is important for apoptotic resistance and constitutive activation of Akt signaling is demonstrated in RCCs [22, 38]. Decreased Akt phosphorylation at late stage was accompanied by a reduction of Akt protein in luteolin-treated 786-O cells. The potential involvement of HSP90 in Akt protein stability and activation was further proved by 17-AAG treatment. Reductions in Akt protein content, Akt phosphorylation, and Ask1 serine-83 phosphorylation were noted in 17-AAG-treated 786-O cells, which were accompanied by an increased phosphorylation of p38 and JNK, and apoptosis. The hypothesis of HSP90 inactivation by luteolin was also supported by the decreased functional complexes between activated Akt and HSP90 and the appearance of proteolytic cleavage of HSP90. These findings suggest the functional importance of HSP90 in Akt protein stability and activity and demonstrate that the inactivation of HSP90/Akt axis is a potential upstream regulator for luteolin-induced Ask1, p38, and JNK activation, which subsequently leads to apoptosis. Protein phosphorylation is governed by kinases and phosphatases. Therefore, the event of phosphatase-mediated dephosphorylation is another crucial step in regulating Akt activity. Evidence shows that PP2A is a selective phosphatase of Akt and protein phosphatase activity could be modulated by luteolin [39, 40]. The involvement of PP2A in luteolin-induced decreased Akt phosphorylation was supported by the elevated activity at the early stage. The reduction of PP2A activity at late stage was possibly due to the cell death. Considering the temporal change of PP2A activity, HSP90 cleavage, Akt phosphorylation, and Akt content, our findings suggest two driving forces critical to luteolin-induced Akt inactivation, PP2A activation at the early stage and HSP90 inactivation at the late stage.

It should be noted that some crucial issues were beyond the scope of this study, such as the detailed mechanisms underlying HSP90 inactivation, Ask1 activation, and p38/JNK-mediated apoptosis. It is hypothesized that Ask1 might be alternatively activated through oxidative stress-induced Ask1 autophosphorylation and activated p38/JNK promotes Bax-dependent apoptosis through Bax phosphorylation. Oxidative stress-dependent HSP90 cleavage occurs in apoptotic cells [29, 30]. However, luteolin-induced HSP90 cleavage may not be the case. The molecular weight of HSP90 proteolytic product in luteolin-treated cells differed from H_2_O_2_-treated cells (data not shown). Some studies have shown that granzyme B, autophagy, and caspase-10 might be potential mediators in the disruption of HSP90 function through proteolytic cleavage [29, 30, 41, 42]. An interesting finding in this study was that the cleavage of HSP90 might also be an apoptosis-associated event caused by luteolin. In spite of the induction of apoptosis, luteolin only caused cleavage of HSP90 in 786-O and A498 VHL-null cells but not ACHN VHL-wild type cells. The cleavage of HSP90 was not detected in LY294002-induced caspase-dependent 786-O cell apoptosis. That is, the cleavage of HSP90 could be mediated by cell-selective and/or stress-selective mechanisms. Since broad-spectrum caspase inhibitor caused nearly complete inhibition of luteolin-induced HSP90 cleavage in 786-O cells, the distinct caspase involved, particularly the caspase-10 is highly expected.

The malignancy and vascularization of metastatic RCC is well associated with the constitutively expressed PI3K/Akt/mTOR axis [38, 43]. mTOR inhibition or PI3K/mTOR dual inhibition as monotherapy for RCC has been proven to be effective, but this may not be the ideal approach because of the negative feedback [15, 44, 45]. Since aberrant cancer-causing pathways address multiple components, a single drug treatment may not be sufficient for long-term control of RCC, either due to the development of resistance or due to the development of compensatory feedback loops. Thus, combination targeted therapy through horizontal blockade or vertical blockade has been the focus of many research efforts in recent years [19, 46]. The role of HSP90 in cell survival and proliferation is well documented; its inhibition has been shown to induce cancer cell apoptosis and improve resistance [29, 36, 37]. However, the precise underlying mechanism of the pathway of cell death and sensitization upon HSP90 inhibition in RCC is not yet fully understood. As with luteolin treatment, pharmacological inhibition of HSP90 or Akt triggered apoptotic execution involving Ask1-p38/JNK signaling. Inhibition of HSP90, Akt, or both aggravated luteolin-induced 786-O cell cytotoxicity and apoptosis. The ability of HSP90 to interact with multiple signaling networks is exploited by cancer cells, in which the expression of HSP90 is increased. Given the number of key nodal proteins that are HSP90 clients, its inactivation represents an interesting target for cancer therapy. Owing to the high expression of HSP90 and the PI3K/Akt/mTOR axis in RCC [38, 43, 47], our findings suggest that the combination of HSP90 inhibitors might be a therapeutic option to improve the efficiency of targeted therapy for RCC.

**Figure 8:**
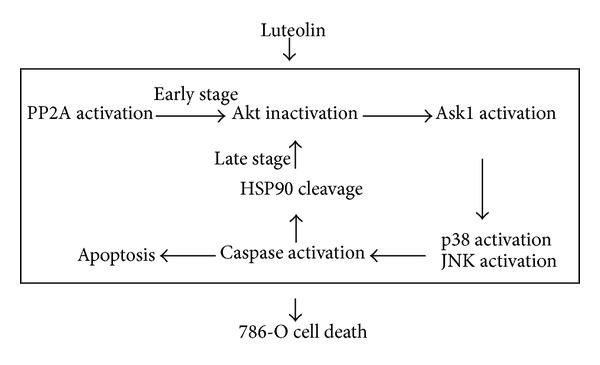
possible signaling cascade of apoptosis elicited by luteolin in 786-O cells is proposed. This schematic diagram indicates the signaling molecules employed in mediating 786-O cell apoptosis after luteolin treatment. Some additional signaling molecules and cascades have been omitted for the sake of clarity.

Plants are a good source of useful health-promoting agents. The health-promoting effects of natural substances originating from plants are the subject of growing interest. There are numerous ongoing efforts to identify novel health-promoting substances, especially from plants with historically documented or pharmacological properties. Among these, flavonoids are a group of polyphenolic compounds that are widely found in the plant kingdom. As intrinsic components of fruits, vegetables, beverages such as wine and tea, and in some traditional herb-containing medicines, many of the different flavonoids known to date are part of the regular human diet. Flavonoids are nonessential dietary factors, but their average daily consumption is estimated to be 1-2 g [48]. A plasma level of 10–20 *μ*M luteolin can be reached in rats after one oral administration at the dose of 50 *μ*moL/kg [49]. Thus, achieving a plasma level of ~40 *μ*M luteolin may not be possible. Alternatively, high-plasma levels of luteolin could be achieved through intravenous injection or repeated administration. A potential advantage of plant-derived compounds in healthcare is that their utilization as food has a long history, and their use has been accepted as safe. The possible utilization of plant-derived compounds and extracts as chemopreventive has spurred interest in research efforts to understand their molecular mechanisms and targets of action. This study provides evidence of an alternative target for luteolin, a dietary flavonoid, and sheds light on the mechanism of its physiological benefits. We have shown that reduction of 786-O RCC cell viability and induction of apoptosis by luteolin may be attributed to the downregulation of Akt and resultant up-regulation of Ask1, p38, and JNK activities, probably via PP2A activation. In addition to being a concurrent substrate of caspases and event of cell death, HSP90 cleavage might also play a role in driving further cellular alterations and cell death, at least in part, involving an Akt-related mechanism (Figure 8). Based on our current understanding of luteolin, it is reasonable to postulate that PP2A, HSP90, Akt, Ask1, p38, and JNK might represent potential therapeutic targets for the treatment of RCC. The antineoplastic mechanism of luteolin seems to be multifactorial. Our findings suggest that PP2A activation might work in concert with HSP90 cleavage to inactivate Akt and lead to a vicious caspase-dependent apoptotic cycle in luteolin-treated 786-O cells.
